# Mitochondrial Permeability Transition Pore: The Cardiovascular Disease’s Molecular Achilles Heel

**DOI:** 10.3390/biomedicines13123014

**Published:** 2025-12-09

**Authors:** Salvatore Nesci, Speranza Rubattu

**Affiliations:** 1 Department of Veterinary Medical Sciences, University of Bologna, 40064 Ozzano Emilia, Italy; 2 IRCCS Neuromed, 86077 Pozzilli, Italy; 3 Clinical and Molecular Department, Sapienza University of Rome, 00189 Rome, Italy

**Keywords:** cardiovascular diseases, mitochondria, myocardial infarction, mitochondrial permeability transition pore, cell death

## Abstract

The mitochondrial permeability transition pore (mPTP) plays a central role in myocardial injury. Upon reperfusion after myocardial infarction, oxidative stress, calcium overload, and ATP depletion promote mPTP opening, leading to mitochondrial dysfunction, cell death, and infarct expansion. This process affects various cardiac cell types differently, contributing to complex pathological remodelling. Key mitochondrial events, such as disruption of bioenergetics parameters, impaired mitophagy, and oxidative stress, drive regulated cell death. Emerging therapies targeting mitochondrial biology, dynamics, and transplantation offer promising strategies to mitigate damage and improve cardiac outcomes. Considering the potential to improve cardiac outcomes and redefine therapeutic approaches in the management of cardiovascular disease, mPTP modulation represents a compelling therapeutic target in myocardial infarction and ischemia–reperfusion injury management.

## 1. Introduction

Cardiovascular diseases (CVDs) remain the leading cause of morbidity and mortality worldwide, with mitochondrial dysfunction increasingly recognized as a pivotal contributor to their pathogenesis. Among the mitochondrial mechanisms implicated in cardiac injury, the mitochondrial permeability transition pore (mPTP) has emerged as a central player in the regulation of cell fate during ischemia–reperfusion events [1]. Upon reperfusion following myocardial infarction, oxidative stress, calcium overload, and ATP depletion converge to trigger mPTP opening, resulting in mitochondrial depolarization, bioenergetic collapse, and activation of regulated cell death pathways.

This mitochondrial crisis affects various cardiac cell types, including cardiomyocytes, endothelial cells, and fibroblasts, differently, contributing to complex pathological remodelling and expansion of the infarct zone. Key mitochondrial processes, such as impaired mitophagy, disrupted dynamics, and excessive reactive oxygen species (ROS) production, further exacerbate tissue damage and compromise cardiac function [2].

Recent advances in mitochondrial biology have unveiled promising therapeutic strategies aimed at modulating mPTP activity, enhancing mitochondrial quality control, and even exploring mitochondrial transplantation. These approaches hold the potential to mitigate myocardial injury, improve cardiac outcomes, and redefine therapeutic paradigms in CVD management. However, challenges remain, including targeted drug delivery, safety, and patient-specific variability. Continued research into mitochondrial-targeted therapies and precision medicine will be essential to translate these insights into effective clinical interventions.

## 2. Background of mPTP

Regulated cell death (RCD) is a maladaptation of cardiomyocytes in heart diseases with various degrees of regulation in multifaceted molecular events [3]. Profound and long-lasting effects of RCD occur in cardiomyocytes, remaining largely refractory to regenerative cues [4]. Cellular energy homeostasis controlled by mitochondria is compromised in the presence of reduced oxygen availability. As a consequence, mitochondrial dysfunction arises from this pathological condition, triggering a cascade of molecular events that compromise cardiac functions [5]. Tissues with high energy demands, such as the heart, suffer bioenergetic failure during hypoxia or ischemic conditions. The alteration of mitochondrial function is caused by the disruption of oxidative phosphorylation (OXPHOS) dependent on oxygen deprivation. Impaired mitochondria can decrease ATP production, whereas mitochondrial calcium homeostasis is often dysregulated, and reactive oxygen species (ROS) are generated by mitochondrial electron transport chain inhibition [6]. This critical triangle promotes deleterious cellular pathology leading to the formation of the mPTP [7]. The collapse of the electrochemical gradient of protons through the inner mitochondrial membrane (IMM) blocks OXPHOS, whereas ions with small molecules efflux-influx across the IMM are not regulated. Uncontrolled water uptake into the mitochondrial matrix, a consequence of mPTP opening, leads to mitochondrial swelling [8]. This swelling causes the outer mitochondrial membrane and/or IMM to rupture to release mitochondrial proteins and causing organelle damage, ultimately resulting in regulated cell death. mPTP formation plays a crucial role in the progression of irreversible damage to cardiovascular cells. The nature of this biological effect has a negative effect on the bioenergetics of mitochondria [9,10]. The permeability transition of the IMM, when an abrupt elevation of intramitochondrial Ca^2^ occurs, is mediated by the opening of mPTP, which permits the passage of solutes with a molecular mass up to approximately 1.5 kDa [11]. The component and structure of mPTP is not fully understood, even if different evidence suggests the adenine nucleotide translocator (ANT) and, in the last 10 years, with strong evidence, the involvement of F_1_F_O_-ATPase in mPTP formation [12,13,14]. Among all the protein components of the mPTP, only cyclophilin D (CyPD) is the protein characterized by having a regulatory role that influences the pore opening [15]. Recently, ATAD3 has been established as the first essential component of the mPTP [16]. Redox state of thiol groups can modulate the formation of mPTP and sulfhydryl reagents, as diamide and phenylarsine oxide are mPTP activators, whereas dibromobimane is an inhibitor [17]. Other amino acids play a key role in mPTP regulation. Protonation of the specific residue of Hys at pH 6.5 has an inhibitory effect on mPTP and can be relieved by carbethoxylation with diethylpyrocarbonate [18]. Conversely, phenylglyoxal, a known compound to cause post-translational modifications of Arg residues, stimulates the mPTP opening [19,20]. Typical inhibitors of mPTP, such as ADP and cations as Mg^2+^, Sr^2+^, Ba^2+^, and Gd^3+^, which are also recognized as substrates and cofactors of F_1_F_O_-ATPase, block mPTP formation [21,22]. Atractylate (ATR) or carboxyatractylate (CATR) and bongkrekate (BKA) are proposed to determine the mPTP opening and mPTP closure, respectively [23]. Moreover, ATR and CATR lock ANT in the c-state (nucleotide binding site facing the cytosol), whereas BKA stabilizes it in the m-state (nucleotide binding site facing the matrix). In addition to this, by considering the natural ligand ATP and ADP of ANT, the adenine nucleotide exerts selective inhibition on the mPTP opening. ANT may, therefore, have a role as a component of mPTP [24]. Pharmacological compounds are used to block the pore opening in pathological conditions. Cyclosporin A is the most commonly used inhibitor of mPTP by targeting the CyPD [25]. However, different small synthetic molecules or natural molecules targeting the F_1_F_O_-ATPase have been proposed in different drug discovery studies to elucidate the mechanisms governing mPTP opening [26,27,28,29,30,31,32,33,34,35,36,37,38].

The conditions used to open the mPTP can selectively activate different pore populations or sensitivities, all characterized by a Ca^2+^-induced channel facilitating Ca^2+^ release. The methods used to detect mPTP activity can make the interpretation of pore formation sensitive to type of assay performed. Direct assessments of mPTP opening include calcium retention capacity and monitoring of transmembrane potential by using fluorescent dyes, mitochondrial swelling assays evaluating optical density linked to osmotic collapse of mitochondria, and electrophysiological measurements detecting the ionic currents dependent on membrane permeability [39].

**Figure 1:**
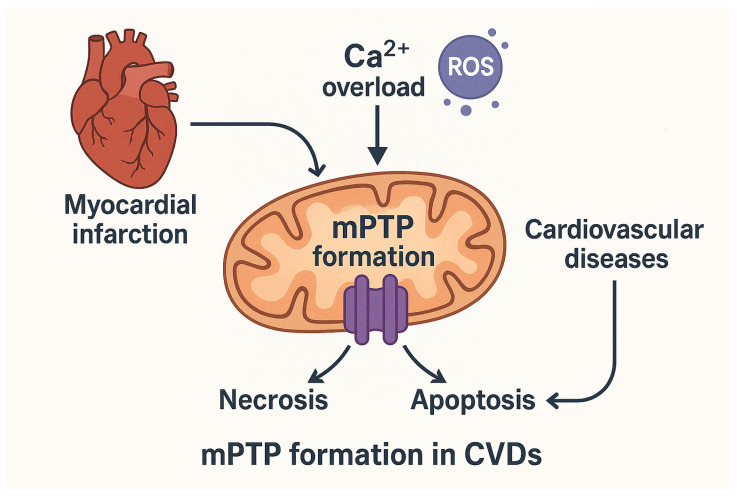
Mitochondrial permeability transition pore (mPTP) opening as a central event in cardiovascular disease (CVD) pathology. Role of mitochondrial Ca^2+^ overload and excessive reactive oxygen species (ROS) production in triggering mPTP opening following myocardial infarction (MI). This event contributes to regulated cell death mechanisms, including necrosis and apoptosis. By mPTP formation, mitochondrial dysfunction and bioenergetic imbalance are key contributors to CVDs progression. The arrows indicate the sequence of biological events. Reactive oxygen species (ROS) are shown as purple circles.

mPTP opening appears to be a pivotal molecular event in the pathological condition, triggering several RCD events following myocardial infarction (MI). For this reason, mPTP desentization is a critical process in ischaemic pre- and postconditioning. Specific inhibition of mPTP opening during reperfusion after acute MI offers significant antinecrotic and antiapoptotic protection [40]. The occurrence and progression of cardiovascular diseases (CVDs) associated with mitochondrial dysfunction are due to a bioenergetics imbalance of mitochondria. Particularly, overload of Ca^2+^ in mitochondria, which disrupts its homeostasis, triggers mPTP opening leading to excessive ROS production, responsible for oxidative stress-induced damage [41,42]. As a consequence of impaired mitochondrial function, the mPTP formation occurs to support pathological changes in the cardiovascular system. The knowledge about the mPTP formation in CVDs is a critical point guiding the development of innovative treatments to improve outcomes and quality of human health (Figure 1). This review provides directions for the mPTP mechanism involved in CVDs as a potential therapeutic target and interventions for the prevention and treatment of mitochondrial dysfunction in CVDs.

## 3. mPTP as a Critical Signalling Axis in Reperfusion Injury

The irreversible damage may occur primarily upon myocardial reperfusion. In this specific context, ischemia/reperfusion (I/R) injury refers to a phenomenon in which the restoration of blood flow may paradoxically result in additional damage. As a protective intervention, ischaemic postconditioning, acting on repeated interruptions of blood flow (ischemia) during the early phase of reperfusion, following a period of sustained ischemia, serves as a protective intervention that can mitigate tissue damage resulting from ischaemia–reperfusion injury [43]. Pharmacological inhibition of the mPTP during reperfusion may serve as a significant adjunct therapy to dramatically reduce infarct size [44]. mPTP opening following acute myocardial infarction is a key event attenuating myocardial apoptosis and necrosis. Cardioprotection has been investigated with a non-immunosuppressive CyPD inhibitor identified as NIM811, which confers cardioprotection when administered at reperfusion. The specific binding to the CyPD, without interaction with cytosolic CyPA, which causes interference within the cellular survival/death pathways, can protect the ischemic heart in in vivo model of acute myocardial infarction [40]. To mitigate lethal reperfusion injury, postconditioning can be used as a potent cardioprotective strategy that relies on brief periods of I/R to limit the damage caused by reperfusion after a prolonged heart attack.

Emerging evidence considers the critical role of mPTP in the mechanism of postconditioning [45]. The complex phenomenon of reperfusion injury encompasses several detrimental biological effects. Prolonged ischemia leads to a progressive breakdown of ionic homeostasis, resulting in intracellular accumulation of Na^+^ and Ca^2+^, ATP depletion, and the onset of ischemic contracture. During ischemia, glycolysis causes a gradual increase in acidosis. Cardiomyocytes counteract the low pH through the Na^+^/H^+^ exchanger, which drives Na^+^ influx. However, due to ATP decreases, Na^+^/K^+^-ATPase cannot regulate intracellular Na^+^ levels. Consequently, the Na^+^/Ca^2+^ exchanger operates in reverse mode, expelling Na^+^ and promoting cytosolic Ca^2+^ overload [45,46,47]. Moreover, Ca^2+^ and Na^+^ play an important role in cells as mediators of second messengers or membrane potential, respectively. Ca^2+^ involved in mPTP opening can be supported by Na^+^ stimulation with ROS production that triggers the mPTP phenomenon. In addition to this, Na^+^ accumulation, behaving like a second messenger, modifies the fluidity of the IMM to control OXPHOS activity and ROS generation [48]. Na^+^ signalling is linked to cell acidification driven by glycolytic activation during ischemia. The resulting decrease in mitochondrial matrix pH triggers the release of free Ca^2+^ from calcium phosphate deposits and simultaneously activates the mitochondrial Na^+^/Ca^2+^ exchanger. This exchanger facilitates Na^+^ entry into the matrix through both electroneutral and electrogenic exchange mechanisms, a process governed by the mitochondrial membrane potential under ischemic conditions. Na^+^ excess interacts with phospholipids of IMM, reducing its fluidity. This decrease in membrane fluidity limits the mobility of free ubiquinone between Complex II and Complex III, while leaving its movement within respiratory supercomplexes unaffected [49]. The alteration of electron transport in respiratory complexes leads to the formation of superoxide at Complex III. Therefore, Na^+^ regulates OXPHOS activity and redox signalling, profoundly impacting cellular metabolism under anaerobic conditions [48]. This dysregulation leads to excessive ROS generation and mitochondrial Ca^2+^ overload, ultimately triggering mPTP opening, the main cause of lethal reperfusion injury [50].

In addition to this, postconditioning also emerges as a promising strategy to mitigate lethal reperfusion injury, and accumulating evidence points toward mPTP as a central player in this process. The temporal coincidence between reperfusion and mPTP opening, the modulation of its activity by genetic and/or pharmacological interventions, and its established role in postconditioning collectively support the hypothesis that mPTP inhibition contributes to postconditioning-induced cardioprotection. Future studies should aim to clarify the precise molecular mechanisms linking postconditioning stimuli to mPTP regulation, as this could open new therapeutic avenues for limiting I/R injury [45]. In lethal reperfusion injury, a powerful anti-ischemic protection is provided with post-conditioning blocks of the mPTP [51].

Recent studies have further elucidated the molecular involvement of necroptosis, a regulated form of cell death distinct from apoptosis, as a key contributor to MI and I/R injury. The results highlight its role as a promising target for cardioprotective strategies. Necroptosis is characterized by its pro-inflammatory properties, which worsen fibrosis, myocardial injury, and unfavourable cardiac remodelling. The regulatory proteins eceptor-interacting protein kinase 3 (RIPK3)/mixed lineage kinase domain-like protein (MLKL), which mediate inflammation and cell death, are essential to this process [52]. According to recent data, calcium/calmodulin-dependent protein kinase II (CaMKII), as a substrate for RIPK3, forms together with mPTP a signalling axis depicted as a crucial mechanism in necroptosis. By blocking the RIPK3-CaMKII-mPTP pathway, it may have cardioprotective effects. Therefore, focusing on necroptosis is a potentially effective treatment approach to reduce cardiac damage, improve recovery, and possibly avoid heart failure after MI [53,54].

**Figure 2:**
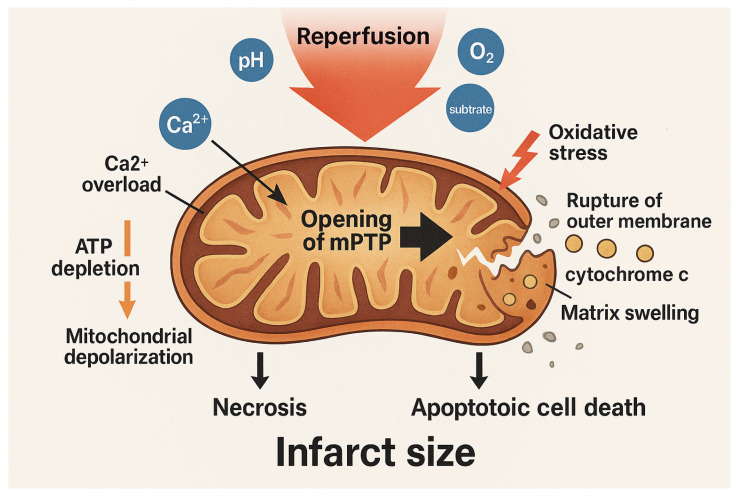
Mitochondrial events during reperfusion injury. Reperfusion restores physiological pH, oxygen, and substrates, triggering a burst of mitochondrial respiration and ROS production. Oxidative stress, combined with calcium overload and ATP depletion, promotes opening of the mitochondrial permeability transition pore (mPTP). mPTP opening causes mitochondrial depolarization, matrix swelling, rupture of the outer membrane, and release of pro-apoptotic factors such as cytochrome c. These events lead to necrotic and apoptotic cell death, contributing to infarct size. The arrows indicate the sequence of biological events. The blue circles represent the different components involved in the reperfusion process. The gold-colored circle indicates cytochrome c.

On balance, upon reperfusion, the abrupt restoration of physiological pH, and oxygen or ischaemic accumulation of succinate [55] triggers a burst of mitochondrial respiration and ROS generation (Figure 2).

This oxidative stress, combined with the pre-existing Ca^2+^ overload and ATP depletion, creates a permissive environment for the opening of mPTP. The mPTP, as a non-specific channel that spans the IMM, when open, leads to mitochondrial depolarization, matrix swelling, rupture of the outer membrane, and release of pro-apoptotic factors such as cytochrome c. These events culminate in necrotic and apoptotic cell death, significantly contributing to infarct size.

## 4. Pathomechanism of Mitochondrial Dysfunction Between Myocardial Cell Types

**Figure 3:**
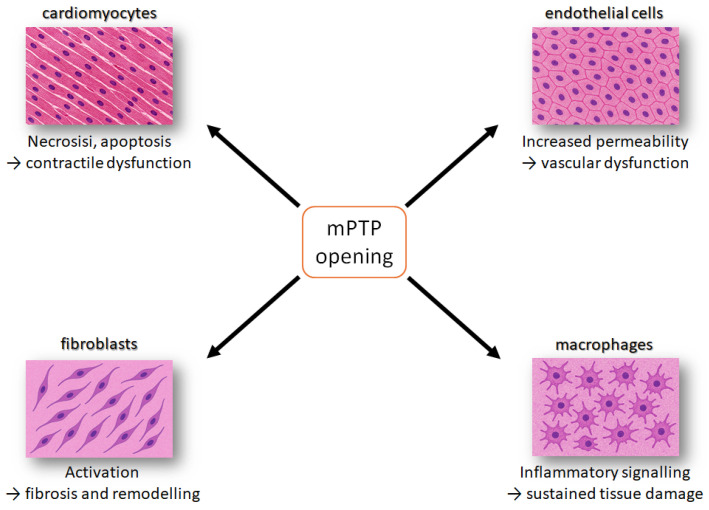
Schematic overview of how mitochondrial permeability transition pore (mPTP) opening affects major cardiac cell types. In cardiomyocytes, mPTP opening induces necrosis and apoptosis, leading to contractile dysfunction. In endothelial cells, it increases permeability, causing vascular dysfunction. In fibroblasts, it promotes activation and extracellular matrix deposition, driving fibrosis and remodelling. In inflammatory cells, it enhances pro-inflammatory signalling, resulting in sustained tissue damage. Together, these pathways contribute to progressive myocardial injury. The orange square indicates the event of mPTP opening, while the arrows refer to the phenomenon occurring in the different cell lines.

Mitochondrial dysfunction in cardiovascular diseases does not affect all myocardial cell types uniformly. The heart is composed of various cell populations, including cardiomyocytes, endothelial cells, fibroblasts, and immune cells, each with distinct metabolic profiles and mitochondrial dependencies. The pathomechanism of mitochondrial dysfunction varies across these cell types, contributing to the complexity of cardiac pathology. Key pathological features, which can be identified in the mPTP opening, Ca^2+^ overload, membrane potential disruption, mitochondrial fission, and impaired mitophagy, drive mitochondrial-dependent regulated cell death, with cell-type-specific manifestations that collectively determine the extent of myocardial injury and remodelling [56] (Figure 3).

### 4.1. Cardiomyocytes

Cardiomyocytes (CMs) rely heavily on mitochondrial OXPHOS to generate ATP required for sustaining contractile function. During ischemia or hypoxia, impaired mitochondrial respiration leads to ATP depletion, Ca^2+^ overload, and production of ROS. Cardiac mitochondrial dysfunction profoundly compromises the essential functions of CMs [56]. Consequently, damaged mitochondria promote the opening of mPTP, resulting in mitochondrial swelling and the release of cytochrome c along with other pro-apoptotic proteins. Alterations in mitochondrial membrane permeability in cardiomyocytes involve Bax-mediated permeabilization of the outer mitochondrial membrane and mPTP-dependent rupture of the IMM, both of which contribute to the initiation of mitochondrial apoptosis [57].

CMs are the primary targets of reperfusion injury following myocardial infarction. During ischemia, ATP depletion and ionic imbalance cause intracellular Ca^2+^ accumulation, predisposing cells to damage upon reperfusion linked to the mPTP phenomenon. Restoration of blood flow triggers a burst of ROS and abrupt pH normalization, which exacerbates oxidative stress and mitochondrial dysfunction. A pivotal event of mPTP opening leads to loss of membrane potential, ATP collapse, and cell death through necrosis or apoptosis. Simultaneously, Ca^2+^ overload combined with renewed ATP availability induces hypercontracture, resulting in contraction band necrosis. Furthermore, cardiomyocytes release damage-associated molecular patterns (DAMPs), amplifying inflammation and contributing to adverse remodelling [58,59]. Collectively, these processes may account for up to 50% of the final infarct size despite successful reperfusion, highlighting the urgent need for targeted cardioprotective strategies.

### 4.2. Endothelial Cells

Compared to cardiomyocytes, vascular endothelial cells (ECs) exhibit greater tolerance to ischemia but are highly susceptible to reperfusion injury, making them key mediators of cardiac I/R damage [60]. ECs play a critical role in supporting cardiomyocyte metabolism and survival through nutrient delivery and the release of paracrine factors such as nitric oxide, ROS, and adenosine, which regulate vascular tone and cellular signalling. Unlike cardiomyocytes, ECs contain relatively few mitochondria (2–6% of cell volume), which serve primarily as signalling hubs rather than major ATP producers. Endothelial mitochondria regulate nitric oxide, ROS, and Ca^2+^ homeostasis under physiological conditions; disruption of these pathways during reperfusion leads to oxidative stress, impaired signalling, and endothelial dysfunction [61]. Similarly to CMs, EC mitochondria undergo persistent dysfunction characterized by altered membrane permeability, release of pro-apoptotic proteins, and activation of caspase cascades, culminating in apoptosis. These events compromise vascular integrity and exacerbate myocardial injury during reperfusion. ECs, which line the coronary vasculature, are less dependent on OXPHOS and more reliant on glycolysis. However, mitochondrial dysfunction in these cells disrupts nitric oxide production, impairs vascular tone, and promotes endothelial activation [2]. This contributes to inflammation, thrombosis, and vascular remodelling. mPTP opening in ECs can exacerbate vascular permeability and leukocyte infiltration, aggravating I/R injury [62].

### 4.3. Cardiac Fibroblasts

Cardiac fibroblasts (CFs) are the most abundant cell type in the heart, accounting for 60–70% of total cells, and play a pivotal role in extracellular matrix production and remodelling. During I/R injury, CFs respond differently from cardiomyocytes and endothelial cells: rather than undergoing extensive apoptosis, they primarily exhibit proliferation and differentiation into myofibroblasts, driving fibrosis and adverse ventricular function [63,64]. Under stress conditions, mitochondrial dysfunction in CFs promotes a metabolic shift toward glycolysis and excessive ROS generation, which further stimulates fibroblast activation and collagen deposition. Although less studied, the involvement of the mPTP in fibroblast activation suggests a potential therapeutic target to limit pathological remodelling. Experimental evidence indicates that mitochondrial signalling regulates CF survival and function: for example, angiotensin II can induce mitochondrial depolarization, increase the Bax/Bcl-2 ratio, and activate caspase-3, leading to apoptosis [65], whereas factors such as osteopontin or trichostatin A can protect CFs. Collectively, these findings highlight the dual role of CF mitochondria in promoting fibrosis and influencing cell fate during post-infarction.

### 4.4. Immune Cells

Resident and infiltrating immune cells, such as macrophages and neutrophils, play a dual role in cardiac I/R injury by orchestrating inflammation and repair [66]. Distinct subpopulations of cardiac innate immune cells, particularly macrophages and neutrophils, have specialized roles in both healthy and diseased hearts. Resident macrophages are essential in maintaining the balance of the immune cardiac homeostasis [67], whereas infiltrating macrophages and neutrophils contribute to tissue damage during I/R injury, followed by roles in tissue repair. Emerging evidence highlights that metabolic pathways critically regulate the phenotypes and functions of these immune cells during cardiac injury. Profiling the metabolic states of innate immune cells, especially resident macrophages, under acute and chronic cardiac conditions offers new insights into cardiac immunometabolism and potential therapeutic strategies [68]. During cardiac injury, these cells undergo metabolic reprogramming, shifting toward glycolysis and altering mitochondrial function. Mitochondrial dysfunction amplifies pro-inflammatory signalling by increasing ROS production and promoting cytokine release. Opening of the mPTP further may exacerbate inflammation and tissue damage through cell death and the release of DAMPs as mitochondrial DNA [69]. Conversely, therapeutic strategies targeting mitochondrial pathways, such as preserving mitochondrial integrity or modulating mPTP activity, hold promise for attenuating inflammation and enhancing cardiac repair.

### 4.5. Intercellular Crosstalk

The interplay between myocardial cell types adds another layer of complexity to the pathomechanism of cardiac injury. Damaged cardiomyocytes release DAMPs, which activate innate immune cells and amplify inflammatory responses [70]. Endothelial dysfunction not only compromises vascular integrity but also promotes fibroblast activation, while fibroblast-derived cytokines and growth factors influence cardiomyocyte survival and remodelling. These reciprocal interactions create a dynamic network of signalling events that drive tissue injury and repair. At the centre of this network lies mitochondrial dysfunction, particularly mPTP opening, which acts as a critical hub linking oxidative stress, Ca^2+^ dysregulation, and cell death pathways across different cell types [71]. This intercellular crosstalk underscores the need for therapeutic strategies targeting mitochondrial signalling to mitigate the progression of cardiac pathologies. Indeed, clinical research could be directed towards therapeutic strategies targeting mitochondrial signalling, particularly the inhibition or modulation of mPTP opening. Such interventions possess the potential to exert pleiotropic effects, mitigating the progression of cardiac pathologies by protecting not only the CMs but also the vascular microenvironment and the inflammatory/fibrotic response [72].

## 5. Mechanism of Mitochondrial Homeostasis and Innovative Treatments to Improve Outcomes and Quality of Life

Mitochondrial homeostasis is essential for maintaining cardiac function, as mitochondria regulate energy production, redox balance, Ca^2+^ signalling, and cell survival. Disruption of these processes leads to the progression of CVDs such as heart failure and I/R injury [73]. This dysregulation extends to core processes of mitochondrial quality control, encompassing dynamic regulation through fusion and fission, maintenance of organelle mass via biogenesis, and turnover through selective clearance via mitophagy. Furthermore, the altered function of mitochondria, as the main cause of ROS production, promotes pro-inflammatory and pro-thrombotic states, where the release of oxidized lipid particles and mitochondrial DNA into the cytoplasm acts as a DAMP, potentially triggering innate immune responses [70,74].

Innovative therapeutic strategies are emerging to restore mitochondrial function and improve patient outcomes. These include pharmacological agents targeting mitochondrial dynamics and bioenergetics, mitochondria-targeted antioxidants, and modulators of mitophagy and mPTP opening [9,58]. Novel approaches such as mitochondrial transplantation, mitochondrial transfer, and gene therapy aim to replace or repair damaged mitochondria, while lifestyle interventions like structured exercise have shown efficacy in enhancing mitochondrial biogenesis and quality control. Collectively, these strategies hold promise for reducing morbidity and improving quality of life in patients with CVDs by targeting the central role of mitochondria in cardiac health [75].

Recent findings highlight novel therapeutic strategies focusing on the modulation of mitochondrial bioenergetics, mitochondrial dynamics and quality control mechanisms, such as mitophagy. However, emerging research has expanded the understanding of mitochondrial-derived signals in cardiac pathophysiology and introduced innovative approaches like mitochondrial transplantation. Transfer therapies of mitochondria may enhance cardiac function after MI and attenuate left ventricular remodelling by reducing fibrosis, limiting apoptosis, and promoting angiogenesis [76]. Nevertheless, several limitations constrain this approach, the most significant being the relatively rapid loss of cardiomyocytes following MI. On balance, targeting mitochondrial pathways represents a highly promising therapeutic strategy for managing CVDs.

However, translating mPTP-targeted interventions into clinical practice remains challenging. Limitations include incomplete understanding of pore structure and regulation in human myocardium, variability in patient response, and potential off-target effects due to the ubiquitous role of mitochondria in multiple tissues. The possibility of disrupting physiological permeability transition, which is crucial in regular Ca^2+^ signalling and metabolic adaptation, raises safety concerns. Furthermore, long-term inhibition of mPTP could predispose to abnormal mitochondrial dynamics or impaired mitophagy. To improve patient stratification, emerging biomarkers such as circulating mtDNA [77], cardiolipin oxidation products, and mitochondrial Ca^2+^ load are being investigated as indicators of mitochondrial stress and therapeutic responsiveness [78]. Addressing these challenges through rigorous preclinical validation and biomarker-driven clinical trials will be essential for successful therapeutic translation.

Beyond these concepts, several mitochondria-directed therapies are advancing from preclinical work into early clinical evaluation (Table 1).

Mitochondria-targeted antioxidants aim to preserve respiratory chain function and limit ROS-driven damage, whereas small molecules that modulate mitochondrial dysfunction, mitophagy, or mPTP opening seek to restore mitochondrial quality control and stress tolerance [29,85]. In parallel, innovative approaches such as mitochondrial transplantation, mitochondrial transfer via extracellular vesicles, and gene- or RNA-based strategies targeting mitochondrial regulators are being explored as means to directly repair or replace dysfunctional organelles [86,87]. Collectively, these emerging interventions could complement guideline-directed therapies and enable more precise, mechanism-based management of CVDs.

## 6. Conclusions

CVDs remain the leading cause of morbidity and mortality worldwide, with mitochondrial dysfunction recognized as a central contributor to their pathogenesis. Recent advances have deepened our understanding of mitochondrial roles in cardiac bioenergetics, signalling, and quality control, while highlighting innovative therapeutic strategies such as modulation of mitophagy and regulated cell death, mPTP-dependent, and mitochondrial-targeted drugs. To build on promising preclinical and early clinical results, it is essential to overcome key challenges, including targeted delivery, safety, and patient variability. Continued research into mitochondrial biology and drug discovery strategies will be essential to develop effective, personalized therapies that improve outcomes and quality of life in patients with CVDs.

## Figures and Tables

**Table 1:** Emerging and under-investigation mitochondria-targeted therapies in CVDs.

Therapeutic Approach	Primary Mitochondrial Target/Mechanism	Evidence/Status in CVD	Ref.
Mitochondria-targeted antioxidants	Scavenges mitochondrial ROS to protect the respiratory chain and cardiolipin	Robust preclinical cardioprotection in I/R models; early clinical studies in vascular and cardiac dysfunction	[79]
Cardiolipin stabilizers/bioenergetic peptides	Stabilize the inner mitochondrial membrane and cardiolipin, improve electron transport and ATP synthesis	Phase II/III trials in I/R injury and primary mitochondrial disease; signals of improved energetics and functional capacity, with mixed outcome data	[80]
mPTP modulators	Inhibit or desensitize the mitochondrial permeability transition pore to prevent necrosis/apoptosis	Strong mechanistic and preclinical rationale	[27,81]
Mitophagy and quality-control enhancers	Promote selective removal of damaged mitochondria and improve mitochondrial turnover	Preclinical cardioprotective effects in I/R and pressure-overload models; early human data mainly from ageing/skeletal-muscle studies	[82]
Mitochondrial transfer/transplantation	Deliver viable, functional mitochondria or mitochondrial components to injured myocardium	Proof-of-concept animal studies and early clinical feasibility reports after cardiac surgery suggest improved contractile recovery and reduced remodelling	[83]
Gene- and RNA-based mitochondrial therapies	Modulate nuclear or mitochondrial genes controlling mitochondrial function, dynamics, or quality control	Currently at preclinical stage; major challenges include targeted delivery, long-term safety, and off-target effects, but offer potential for precision correction of mitochondrial defects	[84]

## Data Availability

Not applicable.

## Abbreviations

The following abbreviations are used in this manuscript:

ANT	adenine nucleotide translocator
ATR	atractylate
BKA	bongkrekate
CaMKII	calcium/calmodulin-dependent protein kinase II;m
CATR	carboxyatractylate
CDVs	cardiovascular diseases
CFs	Cardiac fibroblasts
CMs	Cardiomyocytes
CyPD	cyclophilin D
DAMPs	damage-associated molecular patterns
ECs	endothelial cells
I/R	ischemia/reperfusion
IMM	inner mitochondrial membrane
MI	myocardial infarction
MLKL	mixed lineage kinase domain-like protein
mPTP	mitochondrial permeability transition pore
OXPHOS	oxidative phosphorylation
RCD	Regulated cell death
RIPK3	regulatory proteins eceptor-interacting protein kinase 3
ROS	reactive oxygen species
